# Dysregulation of Circadian Clock Genes as Significant Clinic Factor in the Tumorigenesis of Hepatocellular Carcinoma

**DOI:** 10.1155/2021/8238833

**Published:** 2021-10-29

**Authors:** Youfang Liang, Shaoxiang Wang, Xin Huang, Ruihuan Chai, Qian Tang, Rong Yang, Xiaoqing Huang, Xiao Wang, Kai Zheng

**Affiliations:** ^1^School of Pharmaceutical Sciences, Health Science Center, Shenzhen University, Shenzhen 518060, China; ^2^Shenzhen Key Laboratory for Systemic Aging and Intervention, National Engineering Research Center for Biotechnology (Shenzhen), Medical Research Center, Shenzhen University Health Science Center, Shenzhen 518055, China; ^3^Department of Pharmacy, The Second Clinical Medical College (Shenzhen People's Hospital), Jinan University, Shenzhen, China

## Abstract

Hepatocellular carcinoma (HCC) is the leading cause of cancer-related mortality worldwide due to its asymptomatic onset and poor survival rate. This highlights the urgent need for developing novel diagnostic markers for early HCC detection. The circadian clock is important for maintaining cellular homeostasis and is tightly associated with key tumorigenesis-associated molecular events, suggesting the so-called chronotherapy. An analysis of these core circadian genes may lead to the discovery of biological markers signaling the onset of the disease. In this study, the possible functions of 13 core circadian clock genes (CCGs) in HCC were systematically analyzed with the aim of identifying ideal biomarkers and therapeutic targets. Profiles of HCC patients with clinical and gene expression data were downloaded from The Cancer Genome Atlas and International Cancer Genome Consortium. Various bioinformatics methods were used to investigate the roles of circadian clock genes in HCC tumorigenesis. We found that patients with high *TIMELESS* expression or low *CRY2*, *PER1*, and *RORA* expressions have poor survival. Besides, a prediction model consisting of these four CCGs, the tumor-node-metastasis (TNM) stage, and sex was constructed, demonstrating higher predictive accuracy than the traditional TNM-based model. In addition, pathway analysis showed that these four CCGs are involved in the cell cycle, PI3K/AKT pathway, and fatty acid metabolism. Furthermore, the network of these four CCGs-related coexpressed genes and immune infiltration was analyzed, which revealed the close association with B cells and nTreg cells. Notably, TIMELESS exhibited contrasting effects against CRY2, PER1, and RORA in most situations. In sum, our works revealed that these circadian clock genes *TIMELESS*, *CRY2*, *PER1*, and *RORA* can serve as potential diagnostic and prognostic biomarkers, as well as therapeutic targets, for HCC patients, which may promote HCC chronotherapy by rhythmically regulating drug sensitivity and key cellular signaling pathways.

## 1. Introduction

Liver cancer is the sixth most common type of cancer and the fourth highest cause of cancer-associated death globally [1]. Hepatocellular carcinoma (HCC) accounts for 85–90% of all primary liver cancers with increased incidence and mortality [2]. Although there are several therapeutic treatments of HCC, including surgery, radiotherapy, and chemotherapy, the five-year survival of HCC patients remains low primarily due to the delayed diagnoses [3]. Alpha-fetoprotein (AFP) is a tumor marker commonly used for diagnosing patients with HCC. However, the lack of specificity and accuracy limits its application for early-stage HCC detection. Therefore, it is urgent to search for novel biomarkers to facilitate early detection of HCC and improve the clinical survival rate of HCC patients.

Previous research has demonstrated the link between the circadian clock and key tumorigenesis-associated molecular events [4], suggesting the so-called chronotherapy [5]. The circadian clock is an internal timing system that adjusts behaviors and rhythm according to geophysical time. Similarly, the mammalian circadian clock describes an internal timekeeping mechanism regulating physiology and behavior [6]. A set of core “clock genes” that form a feedback loop of gene transcription and translation has been identified to generate circadian rhythms in cells. The key “positive” transcriptional regulators CLOCK and BMAL1 bind to E-box regulatory elements and transactivate the transcription of the “negative” elements PERs and CRYs, as well as multiple other rhythmically expressed genes.

Conversely, PER and CRY act as repressors to inhibit the CLOCK : BMAL1 complex. Notably, by rhythmically transcriptionally regulating the gene expression and gene activity throughout the genome, circadian clock genes play critical roles in biological processes such as apoptosis, cellular senescence, DNA damage repair, and metastasis [7]. Accumulating evidence has shown the importance of circadian clock genes in the diagnosis, therapy, and prognosis of different kinds of cancers. For instance, the expression alterations of most circadian clock genes were associated with overall survival, tumor-node-metastasis stage, and cellular sensitivity to anticancer drugs [8]. Besides, PER1 and CLOCK were reported as potential biomarkers for head and neck squamous cell carcinoma [9], whereas PER2 was reported to be associated with vital tumor-related genes in oral cancer [10]. Until now, little is known about the roles of circadian clock genes in HCC.

Herein, we systematically characterized the expression pattern of core circadian clock genes, including *ARNTL*, *CLOCK*, *CRY1*, *CRY2*, *DBP*, *NPAS2*, *NR1D1*, *NR1D2*, *PER1*, *PER2*, *PER3*, *RORA*, and *TIMELESS*, and their clinical significances in HCC. The expression and clinical information profiles were extracted from The Cancer Genome Atlas (TCGA) and the International Cancer Genome Consortium (ICGC) databases. Various bioinformatics methods were applied to analyze the data to screen vital hits possibly involved in the development of HCC. We also established a prediction model with high performance to predict the overall survival of HCC patients. Moreover, we comprehensively analyzed the mutation, drug sensitivity, immune infiltration, key cellular signaling pathway, and coexpression network of circadian clock genes in the HCC tumor microenvironment.

## 2. Materials and Methods

### 2.1. Patient Data

The gene expression profiles and clinical information of HCC patients were downloaded from TCGA (https://portal.gdc.cancer.gov/) and ICGC (https://dcc.icgc.org/) databases, containing 50 normal and 374 tumor samples (TCGA) and 202 normal and 240 tumor samples (ICGC), respectively. Univariate and multivariate Cox regression analyses were performed to investigate the correlation between clinicopathological characteristics and overall survival (OS) by R software (4.0.2).

### 2.2. Analysis of Differential Expressed Gene

To investigate the expression difference of circadian clock genes between the tumor and normal samples, 374/424 of tumor samples from TCGA and 240/442 of tumor samples from ICGC were analyzed using the ‘edgeR' package and ‘limma' package, respectively. Log2 fold change (logFC), *P* value, and false discovery rate (FDR) were calculated. Genes with *P* < 0.05 and *FDR* < 0.05 were regarded as differentially expressed genes (DEGs). The expression difference of each gene was shown by boxplots. Besides, a Venn diagram was drawn to show the overlapping genes which represent similar expression tendency in all HCC cases.

### 2.3. Validation of DEGs between HCC and Normal Liver Tissues

Methylation and copy number variation (CNV) analysis were performed to validate the differentially expressed genes between normal liver tissues and tumor tissues. Student's *t*-test was used to analyze the methylation difference between the normal and tumor samples. The correlation between gene CNV and mRNA expression in HCC was also built. A Venn diagram was drawn to present circadian clock genes regulated by both methylation and CNV. The Human Protein Atlas (HPA) (https://www.proteinatlas.org/) database was used to validate the protein expression of DEGs between normal liver tissues and HCC tissues.

### 2.4. Survival Analysis

After dividing patients into the high- and low-expression groups, survival curves were drawn according to the Kaplan-Meier method by ‘survival' package in R software, with significance set at *P* < 0.05. Besides, the receiver operating characteristic (ROC) curves were generated to determine the survival parameters, while the area under the curve (AUC) value determined the prognostic performance of the survival model. In addition, to further verify the result of survival analysis, the hazard ratio (HR) and *P* value of circadian clock genes were calculated through the univariate Cox regression based on the gene expression and overall survival.

### 2.5. Prognosis Prediction Models

Prediction models were used to predict the prognosis of HCC patients based on survival analysis. Through a stepwise multivariate Cox hazard regression analysis, a four-gene model was established. The risk score of each HCC patient was calculated by the following formula:

(1) $$\begin{array}{c} \text{Risk score}=\overset{n}{\underset{i=1}{\sum }}\text{C}\text{oe}f_{i}\times \text{Ex}{\text{p}}_{i,} \end{array}$$

where *n*, Coef, and Exp represent the number of included circadian clock genes, the coefficient of each gene, and the gene expression level, respectively. The ROC curve was then constructed for the cohorts from TCGA and ICGC. The AUC representing the predictability of 3-year survival was also calculated by the ‘survival ROC' package. When the AUC value was >0.6, the prediction method was considered reliable. Furthermore, the HCC patients were grouped into the high-risk and low-risk groups according to the median risk score, and the survival curve was then obtained.

### 2.6. Weighted Gene Coexpression Network Analysis (WGCNA)

WGCNA was performed to construct a gene coexpression network, aimed at finding genes coexpressing with circadian clock genes in HCC tissues. The coexpression network was drawn using Cytoscape software (version 3.8.0).

### 2.7. Immune Infiltrate Analysis

The connection between the gene expression and immune cell infiltration in each sample was evaluated by Immune Cell Abundance Identifier (ImmuCellAI). ImmuCellAI is a database-derived web tool to estimate the abundance of 24 immune cells from gene expression datasets, including RNA-Seq and microarray data, which provides infiltration scores of pancancer.

### 2.8. Pathway Analysis

The potential mechanism of circadian clock genes was explored by Gene Set Cancer Analysis (http://bioinfo.life.hust.edu.cn/web/GSCALite/), which is an online research tool for genomics analysis. A pie chart describes several critical cancer pathways in which the circadian clock genes play different roles. To further determine the underlying mechanism of circadian clock genes, the expression profiles of tumor samples downloaded from TCGA were used to conduct Gene Set Enrichment Analysis (GSEA). Hallmark gene sets (*h*) and Kyoto Encyclopedia of Genes and Genomes gene sets (c2) were used as references. A significant enrichment pathway was used to screen which circadian clock genes were upregulated in the high-risk group, with *P* < 0.05 set as the threshold. Furthermore, drug sensitivity analysis was carried out to investigate the correlation between clock genes and anticancer drugs.

## 3. Results

### 3.1. Circadian Rhythm of Core Circadian Clock Genes in the Liver

**Figure 1:**
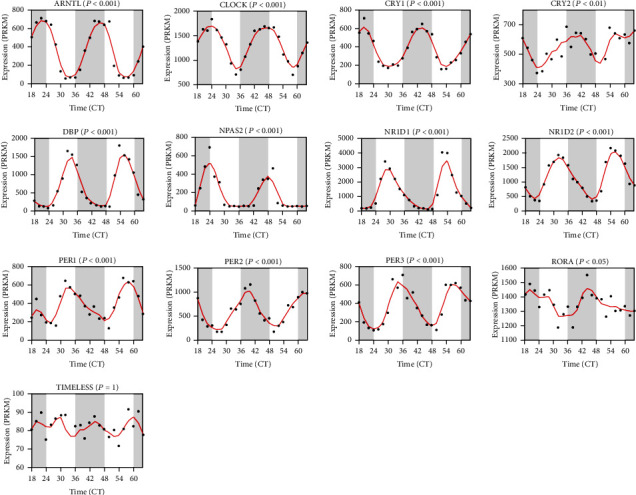
Core circadian clock genes in HCC. The circadian rhythm of core circadian genes in HCC, including *ARNTL*, *CRY1*, *CRY2*, *CLOCK*, *DBP*, *NR1D1*, *NR1D2*, *NPAS2*, *PER1*, *PER2*, *PER3*, *RORA*, and *TIMELESS*. RNA-seq data are from ref. [12].

Herein, we investigated the possible roles of 13 core circadian clock genes in HCC, including *ARNTL*, *CLOCK*, *CRY1*, *CRY2*, *DBP*, *NR1D1*, *NR1D2*, *NPAS2*, *PER1*, *PER2*, *PER3*, *RORA*, and *TIMELESS*. The expression profiles of core circadian genes in liver tissue were explored by RNA sequencing at different intervals [11]. The corresponding expression fluctuations of these genes are shown in Figure 1. Apparently, all these genes showed significant circadian rhythms in liver tissue except *TIMELESS*. Besides, *ARNTL* and *CLOCK*, two central circadian clock regulators controlling the circadian rhythm of *PER*s, *CRY*s, *NR1D*s, *RORA*, *DBP*, and *TIMISS* [6], exhibited the most regular rhythms.

### 3.2. Clinicopathological Characteristics of the HCC Patients

To investigate the functions of circadian clock genes in HCC, 424 samples from TCGA and 442 samples from ICGC were analyzed by univariate and multivariate Cox regression analyses, respectively. In univariate analysis, the poor overall survival of patients was related to tumor-node-metastasis (TNM) stage and T stage in TCGA. It was significantly associated with TNM stage and sex in ICGC (Tables 1 and 2). Clinicopathological characteristics observed with *P* < 0.3 in the univariate analysis were further screened and used for multivariate analysis, revealing that sex and TNM stage might be independent prognostic factors for patients with HCC (Table 2).

### 3.3. Identification of Differentially Expressed Circadian Clock Genes

**Figure 2:**
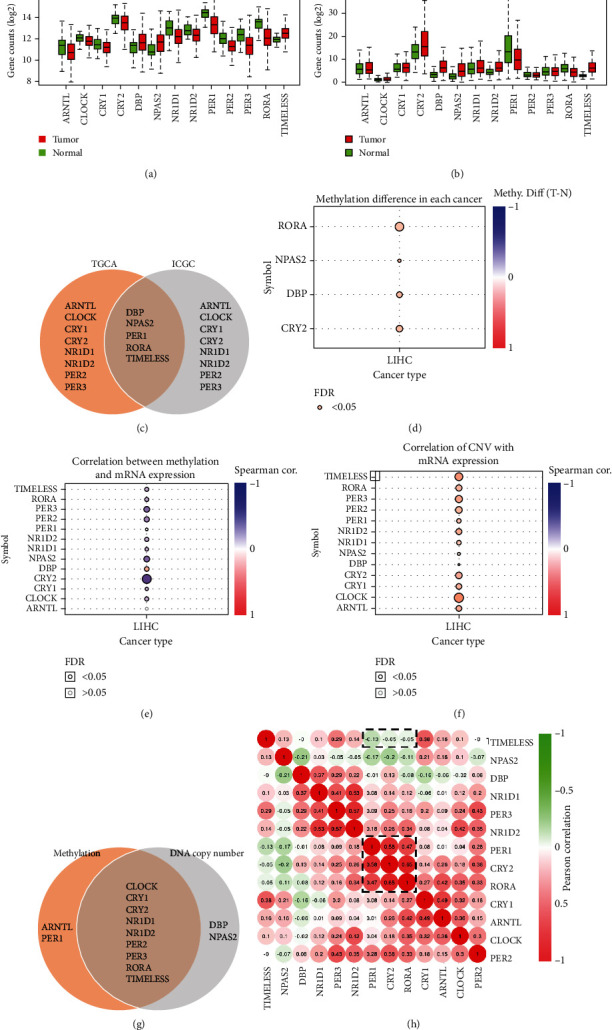
Differential expression analysis of circadian clock genes between HCC and normal tissues. (a, b) Box diagrams showing the expression levels of 13 circadian clock genes in tumor samples compared with normal samples in TCGA and ICGC. The *P* values of the differential expressed four CCGs (*CRY2*, *PER1*, *RORA*, and *TIMELESS*) were >0.05. (c) The circadian clock genes showing a similar expression tendency in TCGA and ICGC. (d) Methylation difference between normal and tumor tissues. (e) Correlation between methylation and mRNA expression. (f) Correlation of copy number variation (CNV) with mRNA expression. (g) Venn diagram showing clock genes that were regulated by both methylation and CNV. (h) The interrelationship between circadian clock genes. TCGA: The Cancer Genome Atlas; ICGC: International Cancer Genome Consortium.

The differential expression of the circadian clock genes between the tumor and normal samples was described using a boxplot (Figures 2(a) and 2(b)). Besides, the overlapping genes that exhibited similar expression levels in tumor samples from both the TCGA and ICGC databases were shown in a Venn diagram, including *DBP*, *NPAS2*, *PER1*, *RORA*, and *TIMELESS* (Figure 2(c)). Next, we analyzed the copy number variation (CNV) and methylation, two important factors influencing the mRNA expression, of these circadian clock genes. As shown in Figure 2(d), the methylation levels of *CRY2*, *DBP*, and *RORA* were statistically higher in HCC tissues than in normal liver tissues. Besides, most of the circadian clock genes were regulated by methylation except for *ARNTL* and *PER1* (Figure 2(e)). The result of the CNV analysis indicated that the mRNA expressions of all circadian clock genes, except for *DBP* and *NPAS2*, were regulated by copy number variation (Figure 2(f)). Moreover, a Venn diagram was drawn to demonstrate that these genes were regulated by both methylation and CNV (Figure 2(g)).

Furthermore, the protein expression levels of *TIMELESS* and *CRY2* were validated using the HPA database. The protein expression level of TIMELESS was increased, and that of *CRY2* was decreased in cancerous tissues compared to those in adjacent noncancerous tissues in HCC patients (Fig. S1), which was in agreement with the bioinformatics analysis. Finally, to investigate the interrelationship between circadian clock genes, the Pearson correlation coefficient was applied to draw the correlation coefficient heatmap based on the gene expression profiles. As shown in Figure 2(h), three circadian clock genes *CRY2*, *PER1*, and *RORA*, were positively and closely related to each other, indicating their similar effects on HCC patients. Additionally, the correlation between each gene was investigated by R software (Fig. S2), which further verified the close relationship between *CRY2*, *PER1*, and *RORA*. On the contrast, *TIMELESS* showed a low relevance to the expression of *CRY2*, *PER1*, and *RORA*, which were slightly negatively associated. Indeed, *CRY2*, *PER1*, and *RORA* were downregulated, and *TIMELESS* was upregulated in tumor tissues, suggesting that *TIMELESS* may play a different role in HCC.

### 3.4. Circadian Clock Genes as Prognostic Biomarkers for HCC Patients

**Figure 3:**
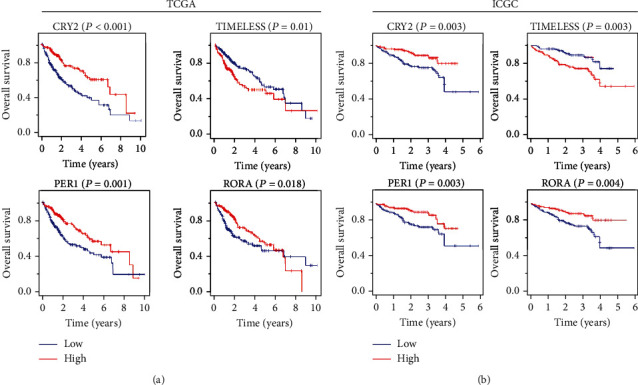
The prognostic value of circadian clock gene in HCC. The role of circadian clock genes in the overall survival of HCC patients based on the TCGA database (a) or ICGC database (b).

HCC patients were grouped into the high- and low-risk groups according to the expression of the targeted gene. The survival curves of circadian clock genes were plotted using the K-M method (Figures 3(a) and 3(b)). Among 13 circadian clock genes, *CRY2*, *PER1*, *RORA*, and *TIMELESS* were the only four genes associated with the overall survival of HCC patients (Fig. S3). Patients with higher *TIMELESS* expression had poorer overall survival rates (*P* = 0.01 in TCGA and *P* = 0.003 in ICGC). On the contrary, patients with lower *CRY2*, *PER1*, and *RORA* expressions exhibited poor overall survival rates (*P* = 0, *P* = 0.001, and *P* = 0.018 in TCGA and *P* = 0.003, *P* = 0.005, and *P* = 0.004 in ICGC, respectively). Collectively, these results suggested that *CRY2*, *PER1*, *RORA*, and *TIMELESS* were closely associated with the prognosis of HCC.

### 3.5. Circadian Clock Gene-Based Prediction Models

**Figure 4:**
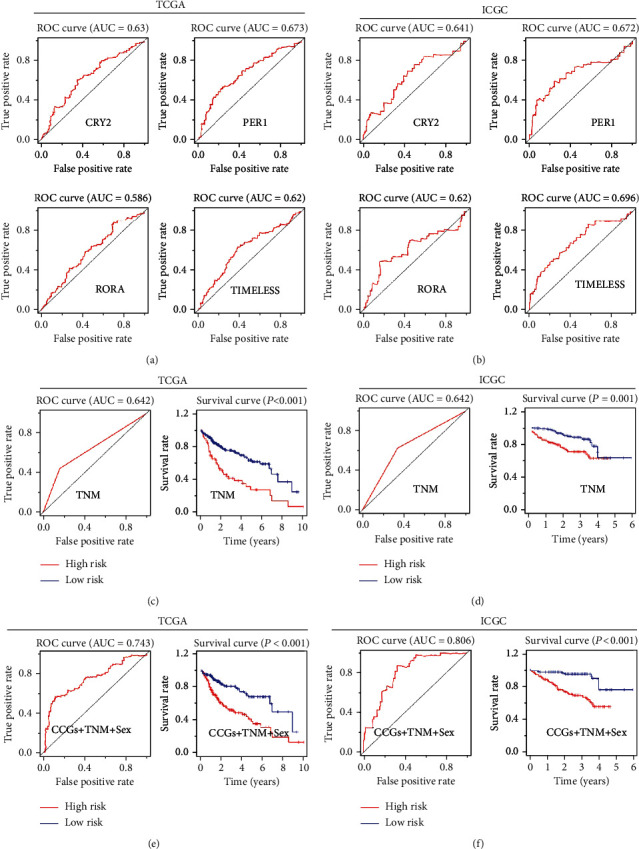
Prediction models to predict the survival of HCC patients. (a, b) ROC and survival curves of single-gene-based models in TCGA and ICGC, respectively. (c, d) ROC and survival curves of TNM stage-based model in TCGA and ICGC, respectively. (e, f) ROC and survival curves of the model consisting of survival-related four genes significantly associated with TNM stage and sex in TCGA and ICGC, respectively. CCGs: the four circadian clock genes.

Subsequently, a circadian clock gene-based prediction model was established to predict patient survival using the multivariate Cox regression analysis. As shown in Figures 4(a) and 4(b), ROC curves of the single-gene model (*CRY2*, *PER1*, *RORA*, and *TIMELESS*, respectively) showed unsatisfactory predictive effects, with the AUC value of 0.6 approximately (0.63, 0.673, 0.586, and 0.62 in TCGA and 0.641, 0.672, 0.62, 0.696 in ICGC, respectively). Furthermore, the traditional TNM stage-based prediction model was constructed, and it was observed that the AUC value was 0.642 in both TCGA and ICGC, which is nearly equal to the single-gene-based model (Figures 4(c) and 4(d)). In addition, the combinatory prediction models consisting of a single circadian clock gene and the TNM stage were constructed, which still exhibited unsatisfactory prediction (Fig. S4). A four-gene-based prediction model combined with two clinicopathological risk factors, TNM stage and sex, was established to further improve predictive frequency (Figures 4(e) and 4(f)). Risk scores of the patients were calculated according to the following formulas:

(2) $$\begin{array}{c} \text{Risk Score }\left(\text{TCGA}\right)=\left(-0.235*\text{CRY}2_{\text{Exp}}\right)+\left(-0.031*{\text{RORA}}_{\text{Exp}}\right)+\left(-0.267*\text{PER}1_{\text{Exp}}\right)+\left(0.077*{\text{TIMELESS}}_{\text{Exp}}\right)+\left(-0.130*\text{SEX}\right)+\left(0.905*\text{TNM}\right),\\ \text{Risk Score }\left(\text{ICGC}\right)=\left(-0.576*\text{CRY}2_{\text{Exp}}\right)+\left(0.193*{\text{RORA}}_{\text{Exp}}\right)+\left(-0.236*\text{PER}1_{\text{Exp}}\right)+\left(0.913*{\text{TIMELESS}}_{\text{Exp}}\right)+\left(-1.109*\text{SEX}\right)+\left(1.135*\text{TNM}\right). \end{array}$$

As a result, the AUC value reached 0.743 in the TCGA database and 0.806 in the ICGC database. Finally, patients were divided into the high-risk and low-risk groups according to the median point, and survival curves were plotted, demonstrating a similar tendency. Collectively, the results showed that the prognostic model proposed in this study effectively predicted the survival of HCC patients.

### 3.6. Nomogram Analysis Indicates the Sampling Time of HCC Patients

**Figure 5:**
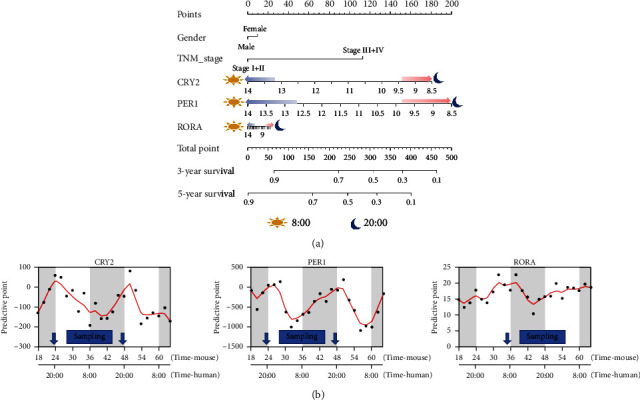
Nomogram analysis showed risk scores in HCC patients. (a) Nomogram based on genes that showed significant circadian rhythms in liver tissue. (b) Detailed display of the predictive point based on gene rhythmic expression.

Furthermore, nomogram analysis was performed based on genes showing significant circadian rhythms in liver tissue, which showed that CCGs, including *CRY2*, *PER1*, and *RORA*, have significant impacts on the predictive accuracy of the 4-CCG-based predictive model (Figure 5(a)). The nomogram results also revealed that lower expression levels of *CRY2*, *PER1*, and *RORA* were associated with higher predictive ability. More importantly, due to the rhythmic expression of CCGs in the liver, the time course of CCG's predictive accuracy was plotted based on their different expression levels (Figure 5(b)). Previous research indicates that the expression peak phase of CCGs shifted by ~12 hours between the mouse and baboon [12]. Accordingly, we found that, when patents sampling at night (8:00 pm), *CRY2* and *PER1* reached their peak, resulting in higher risk scores and facilitating the early diagnosis of patients. Therefore, it is better to sample the HCC patients in the evening to obtain a more accurate predictive function.

### 3.7. Molecular Mechanisms of Circadian Clock Genes in HCC

**Figure 6:**
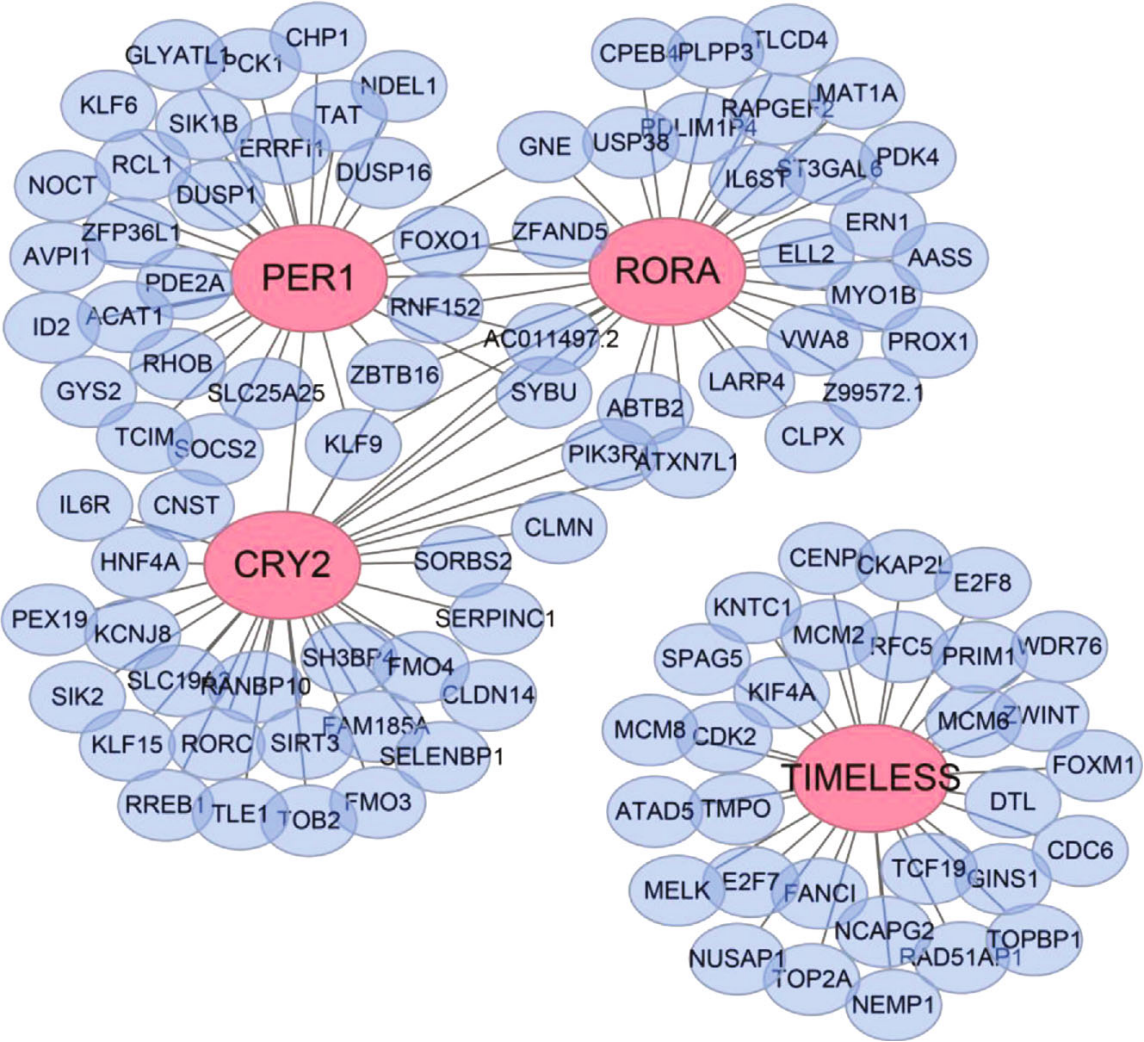
Coexpression network of circadian clock genes. The red nodes are circadian clock genes, while the blue nodes are the coexpressed genes.

To investigate the underlying mechanisms of circadian clock genes in the prognosis and diagnosis of HCC, firstly, WGCNA was performed to construct a coexpression gene network of the four core clock genes. As shown in Figure 5, these four clock genes are marked as large red nodes, whereas blue nodes represent the other coexpressed genes. Notably, gene *CRY2*, *PER1*, and *RORA* were closely associated, possessing several mutual cooperators (hereafter referred to as Cluster 1). However, *TIMELESS* was a relatively independent part of the coexpression gene network (Figure 6). This result was in accordance with the interrelationship between circadian clock genes (Figure 2(h)).

**Figure 7:**
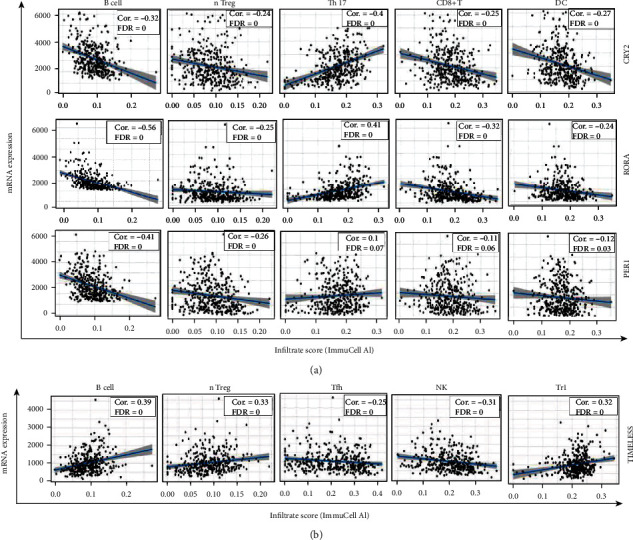
The correlation between circadian clock genes and the immune infiltration level in HCC. nTreg: natural regulator T cell; Th17: T helper 17 cells; DC: dendritic cell; Tfh: T follicular helper cell; NK, natural killer cell; Tr1: type 1 regulatory T cell.

Previous studies have revealed the connection between the circadian rhythm and tumor microenvironment [13]. However, the role of the circadian clock in the tumor microenvironment remains unclear. Next, a correlation analysis was performed between the four core circadian clock genes and the infiltration levels of different immune cells (Figure 7). It was observed that Cluster 1 was significantly negatively associated with B cell, natural CD4+ regulatory T cell (nTreg), CD8+ T cell, and dendritic cell (DC) and positively related with the infiltration of T helper 17 (Th17) cell. On the contrary, *TIMELESS* was positively associated with B cell and nTreg cell. *TIMELESS* was also correlated with Tfh cell, NK cell, and Tr1 cell. These results indicated that Cluster 1 and *TIMELESS* might affect the survival of HCC patients by regulating immune infiltration levels, especially B cell and nTreg cell.

**Figure 8:**
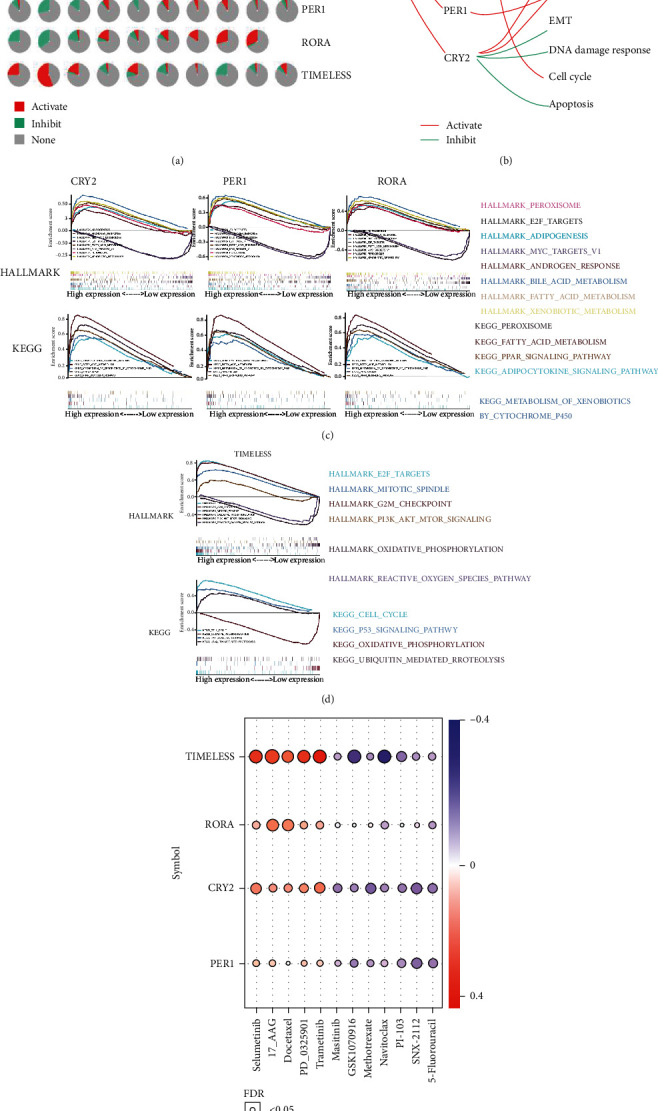
Pathway associated with survival-related clock genes. (a, b) Correlation between survival-related genes and cancer-related pathways. Pancancer (a) or liver cancer analysis (b) was performed to find the key cellular processes associated with the four CCGs. (c, d) GSEA-enriched terms. (e) Drug sensitivity of four survival-related genes.

In addition, the role of circadian clock genes in cancer-related signaling pathways, including TSC/mTOR, RTK, RAS/MAPK, PI3K/AKT, hormone ER, hormone AR, EMT, DNA damage response, cell cycle, and apoptosis pathways, were examined (Figures 8(a) and 8(b)). As shown in pancancer analysis (Figure 8(a)) or liver cancer analysis (Figure 8(b)), Cluster 1 and *TIMELESS* exerted opposite effects on the same signaling pathway; that is, Cluster 1 activated, whereas *TIMELESS* inhibited the same pathway and vice versa. Besides, Cluster 1 mainly inhibited apoptosis, cell cycle, and DNA damage response, which play a critical role in maintaining uncontrolled proliferation and chemoresistance of cancer cells. For a better understanding of the molecular functions underlying the oncogenesis of early HCC, Gene Set Enrichment Analysis (GSEA) was performed, which showed that each clock gene of Cluster 1 was enriched in the same pathway, such as fatty acid metabolism, adipogenesis, bile acid metabolism, and peroxisome pathway based on the Hallmark Gene Sets. By contrast, *TIMELESS* was involved in pathways, including mitotic spindle, oxidative phosphorylation, and the E2F pathway. KEGG gene sets were also applied as a reference cohort (Figures 8(c) and 8(d)). It was also observed that Cluster 1 was closely positively related to the metabolism of amino acids, whereas *TIMELESS* was related to DNA replication and DNA repair-associated signaling pathways (Fig. S5).

Cancer chronotherapy, a therapeutic treatment at a specific time following circadian rhythms, may improve the antitumor effects and reduce toxicity [14]. Accordingly, the correlation between clock gene expression and drug sensitivity was also investigated using datasets from Genomics of Drug Sensitivity in Cancer (GDSC), in which high expression means resistance to a particular anticancer drug. We found that higher expression of Cluster 1 exhibited a similar positive correlation with chemoreagents such as selumetinib, 17-AAG, docetaxel, PD-0325901, and trametinib. Conversely, *TIMELESS* showed a stronger negative correlation with masitinib, GSK1070916, methotrexate, navitoclax, PI-103, SNX-2112, and 5-fluorouracil (Figure 8(e)). These results suggested that inhibition of Cluster 1 or activation of TIMELESS might enhance the chemotherapeutic sensitivity toward special anticancer drugs.

## 4. Discussion

This study demonstrated that four circadian clock genes, including *CRY2*, *PER1*, *RORA*, and *TIMELESS*, could be potential diagnostic and prognostic biomarkers for HCC patients. We also established a prediction model consisting of these four genes, TNM stage, and sex, demonstrating high predictive ability. In addition, it was shown that Cluster 1 (*CRY2*, *PER1,* and *RORA*) and *TIMELESS* exerted opposite impacts on interactive gene network, infiltration of immune cells, cancer-related signaling pathways, and cellular sensitivity to clinically used drugs.

Disruption of the circadian rhythm always leads to physiological disorders of homeostasis in mammals, which is closely associated with the development of cancer [4]. Gene expression, cell cycle, and DNA repair are regulated by the clock genes, providing the base to the hypothesis that disruption of biorhythms may predispose individuals to cancer [6]. Considering the possibility that circadian clock genes play a pivotal role in the physiological functions of mammals, rendering individuals towards the development of cancer [15], the differential expression of core circadian clock genes between HCC tissues and normal tissues was discussed. It was observed that *DBP*, *NPAS2*, *PER1*, *RORA*, and *TIMELESS* showed similar expression tendency in HCC tissues in the TCGA and ICGC databases. The mRNA expression was either affected by methylation [16] or by copy number variation (CNV), and the fluctuation of DNA copy number was found responsible for the alteration in coding RNA expression level [17]. It was also observed that the expression of genes such as *CRY2*, *DBP*, *NPAS2*, and *RORA* was significantly affected by methylation (Figure 2(d)), and all circadian clock genes, except for *DBP* and *NPAS2*, exhibited a significant correlation with CNV. Collectively, the results mentioned above implied the involvement of methylation or CNV in the dysregulation of circadian clock genes.

In addition, we demonstrated that the dysregulation of circadian clock genes was associated with the prognosis of HCC patients. High expression of Cluster 1 (*CRY2*, *PER1*, and *RORA*), or low expression of *TIMELESS*, was correlated with prolonged overall survival (OS) of patients (Figure 3). The investigation of the molecular mechanisms revealed that Cluster 1 and *TIMELESS* counteractively regulated the infiltration of several immune cells such as B cells and nTreg cells. Inherently, B cells can inhibit tumor growth by producing antibodies and presenting tumor antigens, while nTreg cells control the inflammatory microenvironment to restrict tumor development [18–20]. High expression of Cluster 1, or low expression of *TIMELESS*, might inhibit both the infiltration of B cells and nTreg cells (Figure 7), suggesting that the dysregulation of circadian clock genes may manifest HCC by disrupting the tumor microenvironment.

Another important finding of this study was that dysregulation of the circadian clock genes was also found to be associated with several cancer-related pathways (Figure 8), such as DNA damage response, cell cycle, and apoptosis, which is in accordance with previous research that the circadian clock genes influenced cancer susceptibility through DNA damage and apoptosis [21]. Although the cell cycle and circadian clock genes are considered two different biological oscillators, their close relation and interaction have been reported [22]. The GSEA results showed that gene sets of E2F targets, fatty acid metabolism, AKT/mTOR, and p53 signal pathway were significantly enriched. Similarly, Cluster 1 and *TIMELESS* exerted effects on these signaling pathways conversely. Moreover, AKT/mTOR and p53 pathways played vital roles in regulating cell proliferation, and *TIMELESS* could promote the proliferation of HCC cells by inhibiting the p53-dependent signals [23], affirming the finding that high expression of *TIMELESS* is related to poor survival of HCC patients (Figure 3).

Furthermore, the interaction between the circadian clock genes and cellular sensitivity to an anticancer drug was analyzed. Several chemoreagents, such as 5-FU [24] and docetaxel [25], have demonstrated potent antiliver cancer activities. It was observed that higher expression of Cluster 1 might enhance the chemoresistance of these anticancer reagents, implying that inhibition of Cluster 1, or activation of *TIMELESS*, may render liver cancer cells more sensitive to chemotherapy.

## 5. Conclusion

This work demonstrated that four CCGs, including *CRY2*, *PER1*, *RORA*, and *TIMELESS*, could be potential diagnostic and prognostic biomarkers for HCC patients. Besides, *CRY2*, *PER1*, and *RORA* exerted opposite impacts against *TIMELESS* on immune cell infiltration and cancer-related signaling pathways, affecting the overall survival of HCC patients. Selective regulation of circadian clock genes may further assist in precise chronotherapy of HCC patients.

## Figures and Tables

**Table 1:** Univariate and multivariate analyses of clinicopathological characteristics for overall survival in HCC patients from the TCGA dataset (*N* = 318).

Variables	*n* (%)	Univariate analysis		Multivariate analysis	
		HR (95% CI)	*P*	HR (95% CI)	*P*
Age					
<60	152 (47.8%)	1 (reference)			
>60	166 (52.2%)	1.173 (0.796-1.730)	0.421		
Sex					
Female	99 (31.1%)	1 (reference)		1 (reference)	
Male	219 (68.9%)	0.804 (0.539-1.198)	0.284	0.864 (0.579-1.287)	0.472
TNM stage					
I+II	237 (73.9%)	1 (reference)		1 (reference)	
III+IV	83 (26.1%)	2.815 (1.909-4.151)	<0.001	1.522 (0.206-11.219)	0.68
Tumor grade					
G1+G2	197 (61.9%)	1 (reference)			
G3+G4	121 (38.1%)	1.077 (0.724-1.603)	0.713		
T stage					
T1+T2	237 (74.5%)	1 (reference)		1 (reference)	
T3+T4	81 (25.5%)	2.839 (1.923-4.189)	<0.001	1.822 (0.247-13.464)	0.556

Note: characteristics with *P* < 0.3 in the univariate analysis were further screened in the multivariate analysis. HR: hazard ratio; CI: confidence interval; TNM stage: tumor-node-metastasis stage; T stage: stage of tumor invasion.

**Table 2:** Univariate and multivariate analyses of clinicopathological characteristics for overall survival in HCC patients from the ICGC dataset (*N* = 231).

Variables	*n* (%)	Univariate analysis		Multivariate analysis	
		HR (95% CI)	*P*	HR (95% CI)	*P*
Age					
<60	44 (19.0%)	1 (reference)			
>60	187 (81.0%)	0.890 (0.426-1.862)	0.758		
Sex					
Female	61 (26.4%)	1 (reference)		1 (reference)	
Male	170 (73.6%)	0.502 (0.268-0.940)	0.031	0.389 (0.203-0.744)	0.004
TNM stage					
I+II	141 (61.0%)	1 (reference)		1 (reference)	
III+IV	90 (39.0%)	2.492 (1.351-4.599)	0.003	3.003 (1.598-5.645)	<0.001

Note: characteristics with *P* < 0.3 in the univariate analysis were further screened in the multivariate analysis. HR: hazard ratio; CI: confidence interval; TNM stage: tumor-node-metastasis stage.

## Data Availability

The datasets presented in this study, including TCGA and ICGC, can be found online.

## Abbreviations

HCC: Hepatocellular carcinoma
PLC: Primary liver cancer
TCGA: The Cancer Genome Atlas
ICGC: International Cancer Genome Consortium
logFC: Log_2_foldchange
FDR: False discovery rate
DEGs: Differentially expressed genes
CNV: Copy number variation
HR: Hazard ratio
WGCNA: Weighted gene coexpression network analysis
GSEA: Gene Set Enrichment Analysis
TNM stage: Tumor-node-metastasis stage.
