# Supplementary material for: Dysregulation of Circadian Clock Genes as Significant Clinic Factor in the Tumorigenesis of Hepatocellular Carcinoma
Source: Comput Math Methods Med. 2021 Oct 29;2021:8238833. doi: 10.1155/2021/8238833 (PMC8570900; doi:10.1155/2021/8238833)
Supplement: Supplementary Materials — Supplementary material containing four figures is available on the publisher's website along with the published article. [file 8238833.f1.zip › FigS5.pdf]

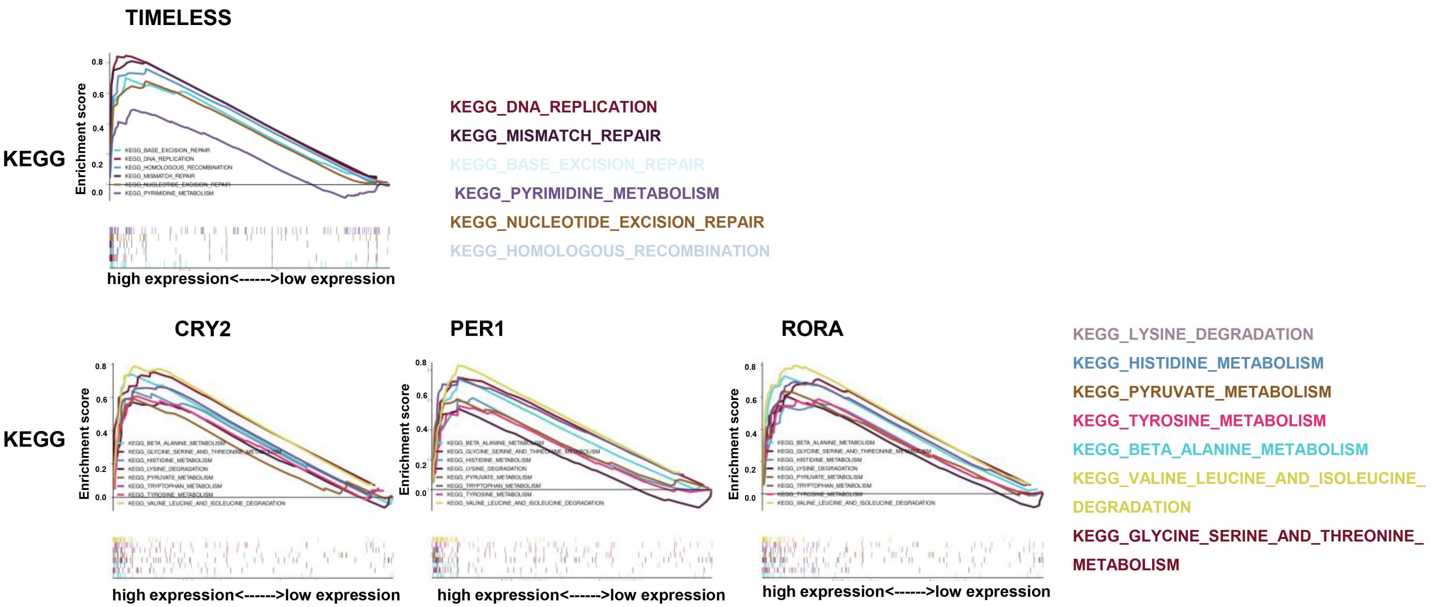
TIMELESS
KEGG
Enrichment score
0.8
0.6
0.4
0.2
0.0
high expression<------>low expression
KEGG_DNA_REPLICATION
KEGG_MISMATCH_REPAIR
KEGG_BASE_EXCISION_REPAIR
KEGG_PYRIMIDINE_METABOLISM
KEGG_NUCLEOTIDE_EXCISION_REPAIR
KEGG_HOMOLOGOUS_RECOMBINATION
CRY2
PER1
RORA
KEGG
Enrichment score
high expression<------>low expression
high expression<------>low expression
high expression<------>low expression
KEGG_LYSINE_DEGRADATION
KEGG_HISTIDINE_METABOLISM
KEGG_PYRUVATE_METABOLISM
KEGG_TYROSINE_METABOLISM
KEGG_BETA_ALANINE_METABOLISM
KEGG_VALINE_LEUCINE_AND_ISOLEUCINE_
DEGRADATION
KEGG_GLYCINE_SERINE_AND_THREONINE_
METABOLISM
