# Supplementary material for: Dysregulation of Circadian Clock Genes as Significant Clinic Factor in the Tumorigenesis of Hepatocellular Carcinoma
Source: Comput Math Methods Med. 2021 Oct 29;2021:8238833. doi: 10.1155/2021/8238833 (PMC8570900; doi:10.1155/2021/8238833)
Supplement: Supplementary Materials — Supplementary material containing four figures is available on the publisher's website along with the published article. [file 8238833.f1.zip › Supplemental information (1).docx]

**Supplemental information**

**Deregulation of circadian clock genes as significant clinic factor in the tumorigenesis of Hepatocellular carcinoma**

**Supplemental figures and legends**


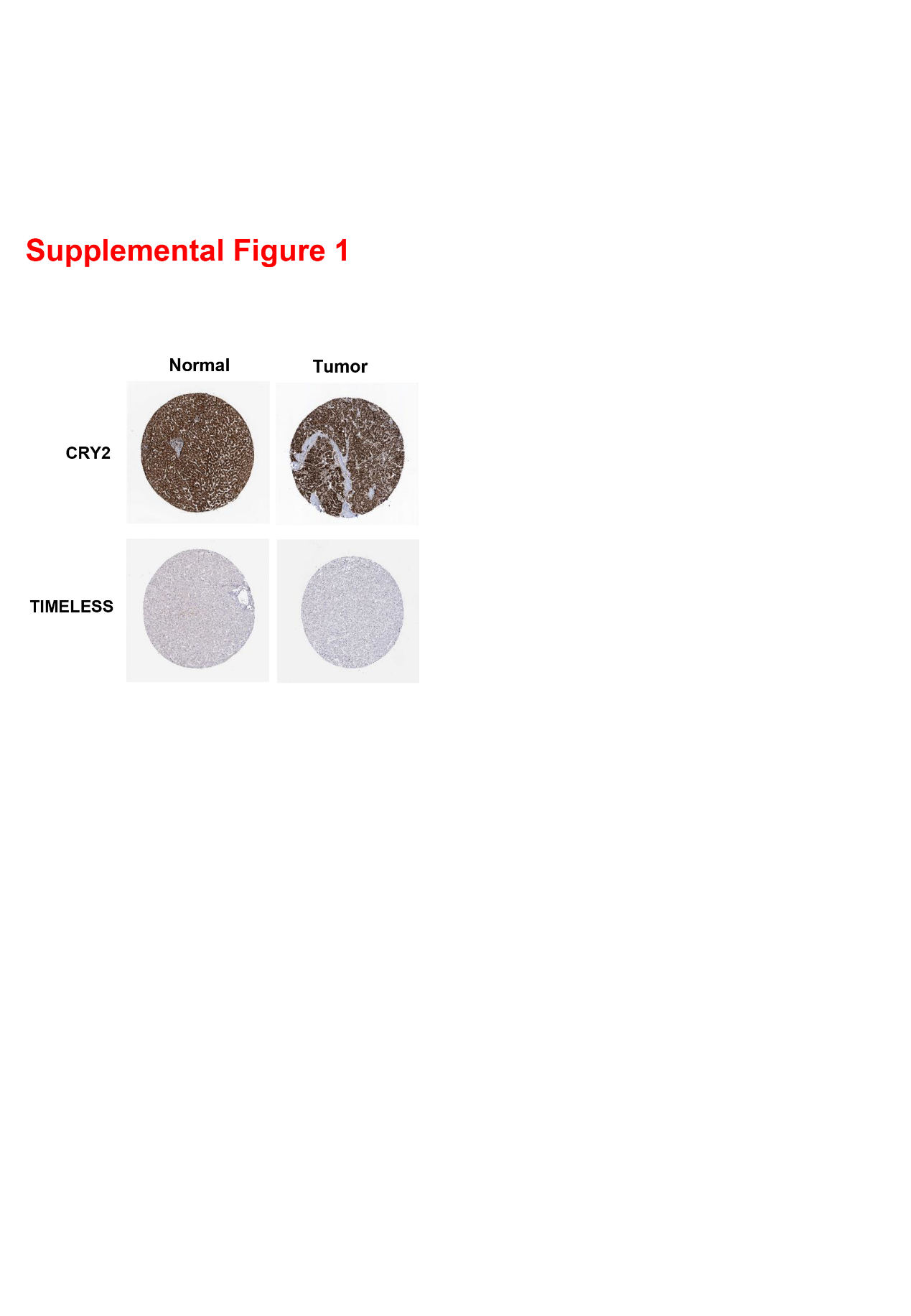


**Figure S1. Immunohistochemical analysis of CRY2 and TIMELESS in HCC tissues and normal tissues.**


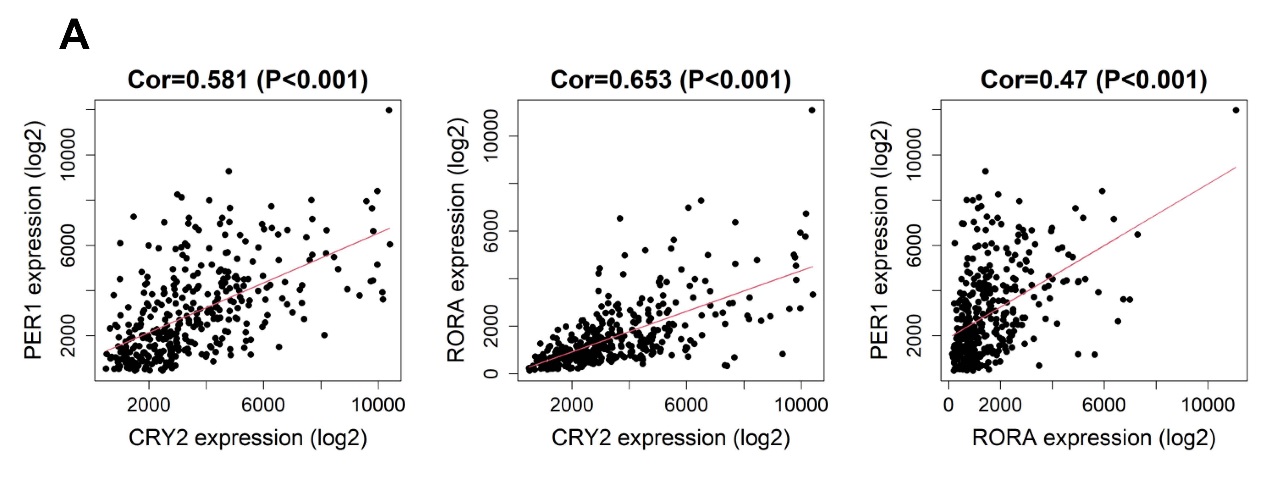


**Figure S2. Expression correlation between PER1, CRY2 and RORA in liver cancer.**


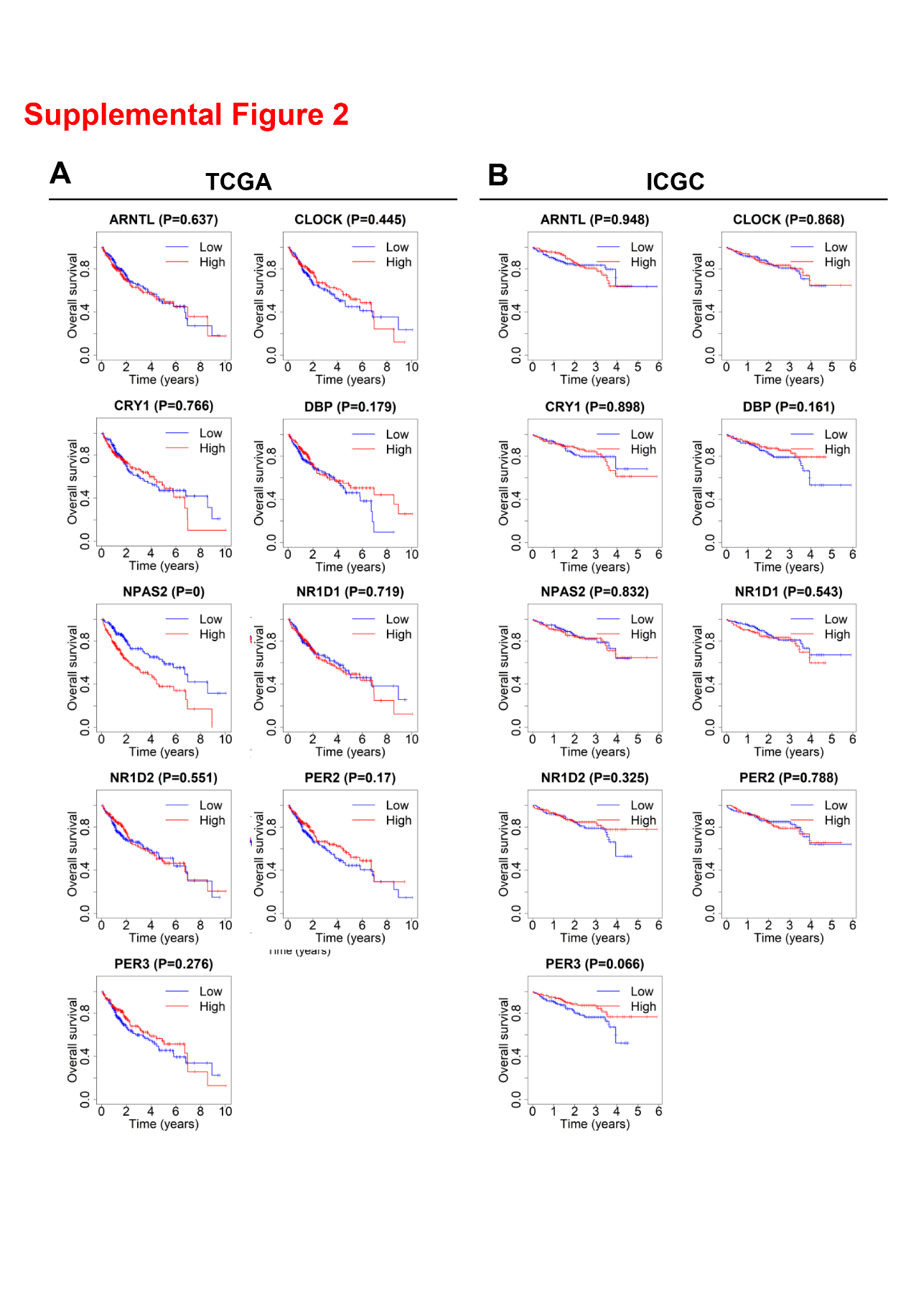


**Figure S3. The role of circadian clock genes in the overall survival of HCC patients based on TCGA and ICGC database.**


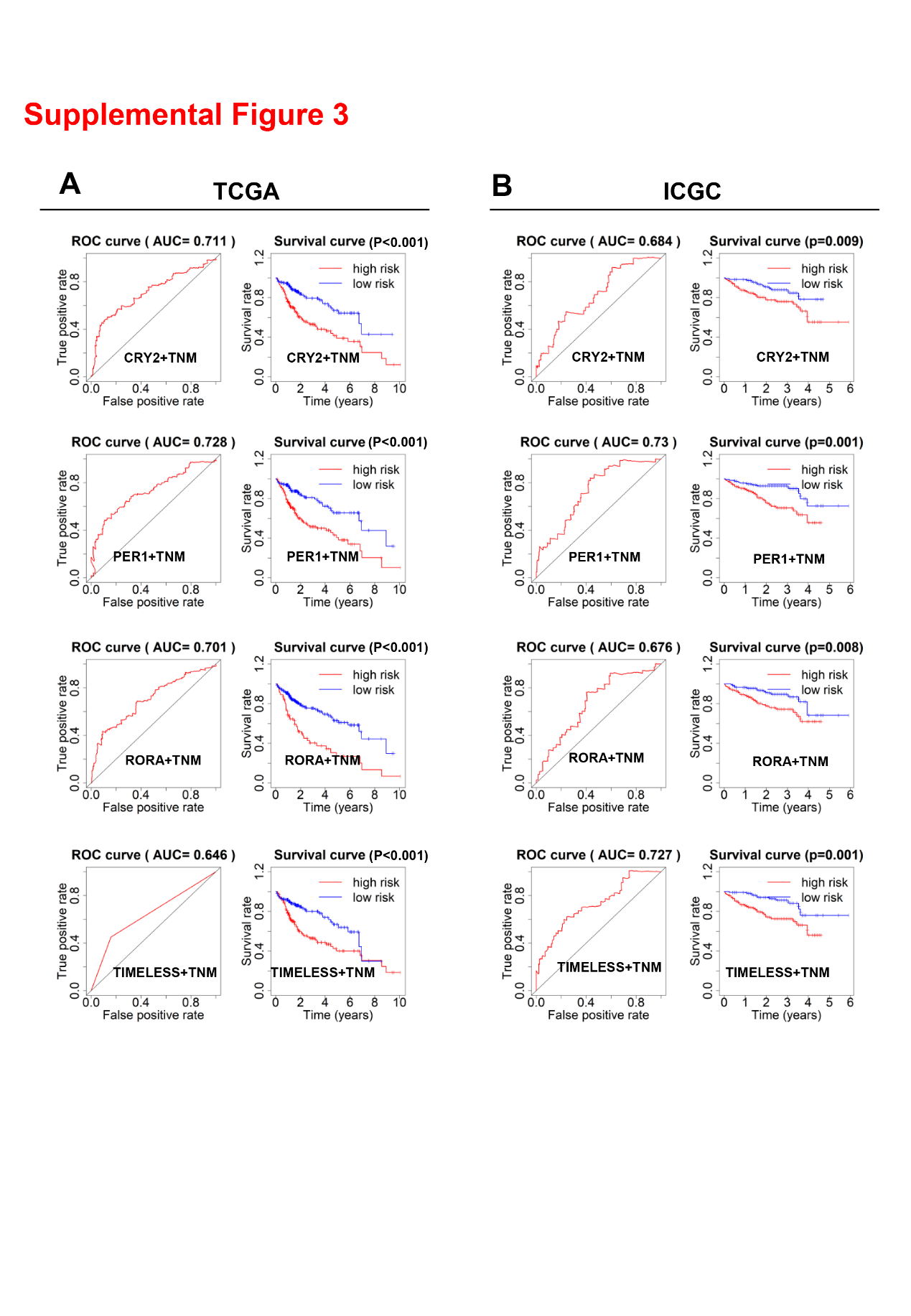


**Figure S4. Prediction models to predict the survival of HCC patients.** Prediction model based on single circadian clock gene and TNM stage was constructed, and the corresponding survival curve was also plotted.


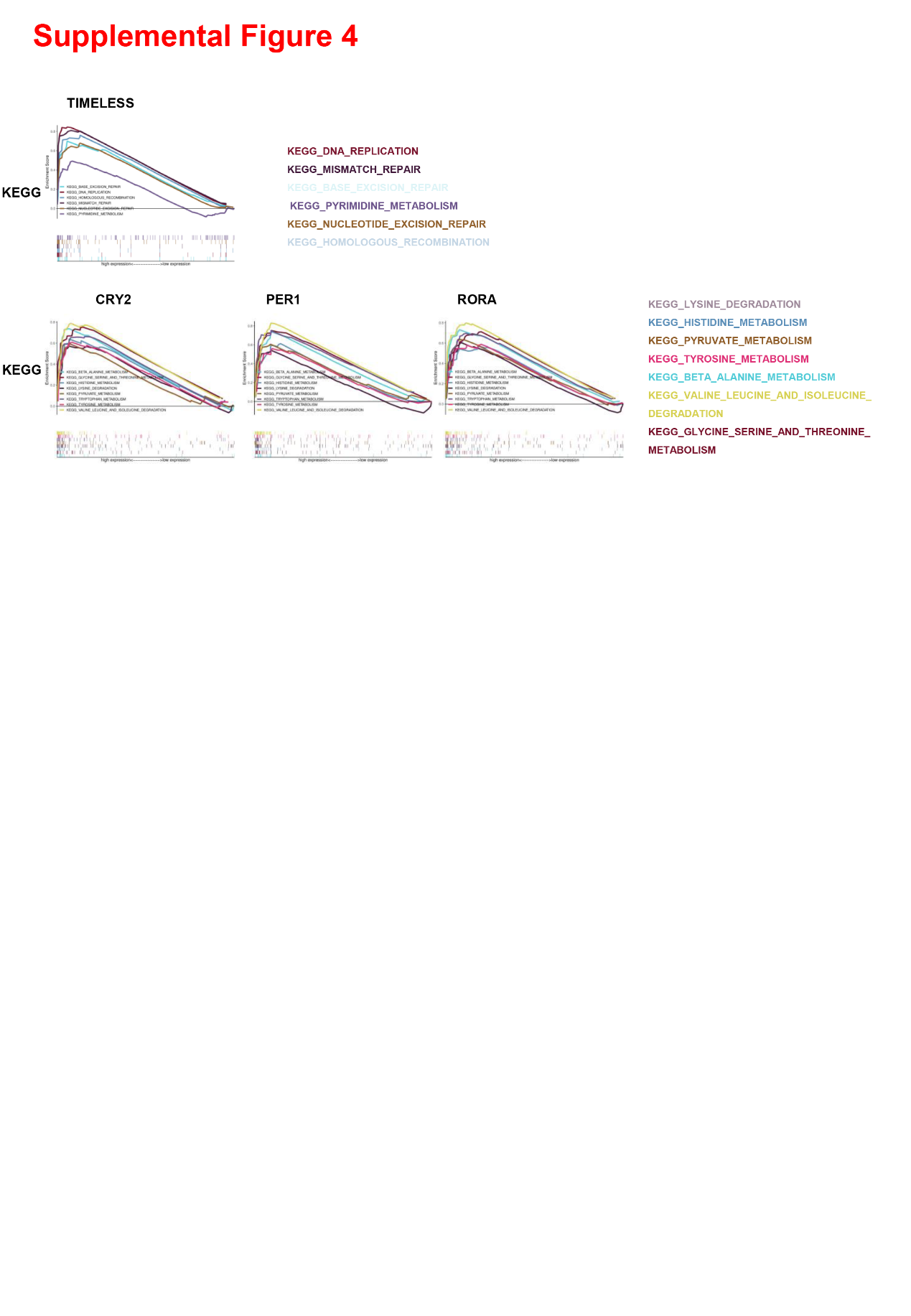


**Figure S5. GSEA enriched terms.**
