# Supplementary figures and images for: Dysregulation of Circadian Clock Genes as Significant Clinic Factor in the Tumorigenesis of Hepatocellular Carcinoma
Source: Comput Math Methods Med. 2021 Oct 29;2021:8238833. doi: 10.1155/2021/8238833 (PMC8570900; doi:10.1155/2021/8238833)

**Normal**

**Tumor**

**CRY2**

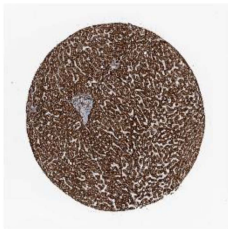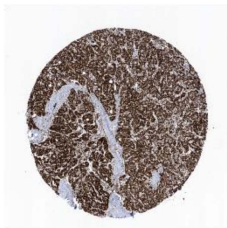

**TIMELESS**

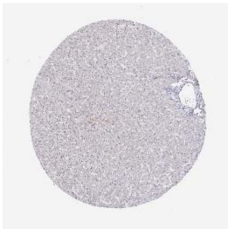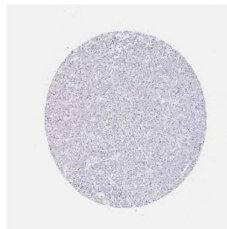

Supplement: Supplementary Materials — Supplementary material containing four figures is available on the publisher's website along with the published article. [file 8238833.f1.zip › FigS1.pdf]

**A****Cor=0.581 (P<0.001)**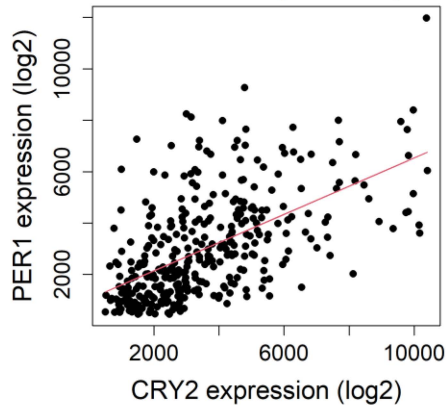**Cor=0.653 (P<0.001)**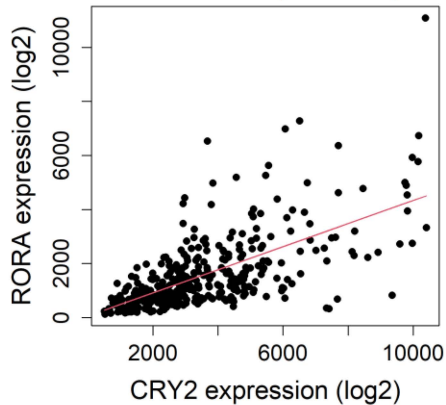**Cor=0.47 (P<0.001)**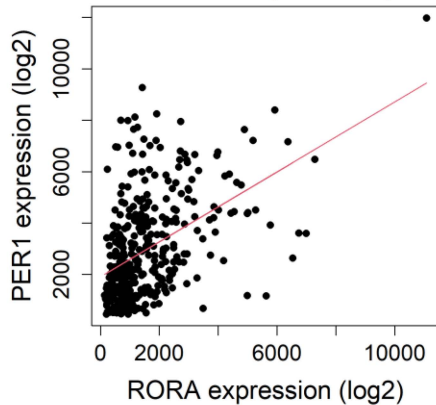

Supplement: Supplementary Materials — Supplementary material containing four figures is available on the publisher's website along with the published article. [file 8238833.f1.zip › FigS2.pdf]

**A****TCGA**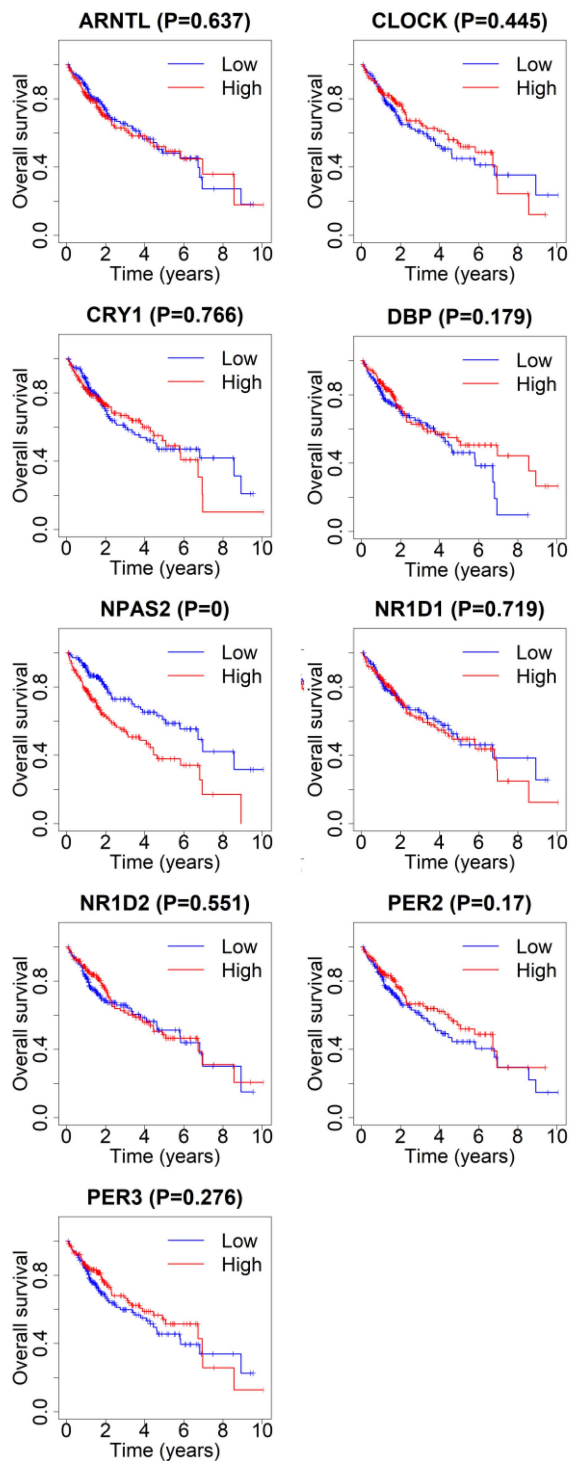**B****ICGC**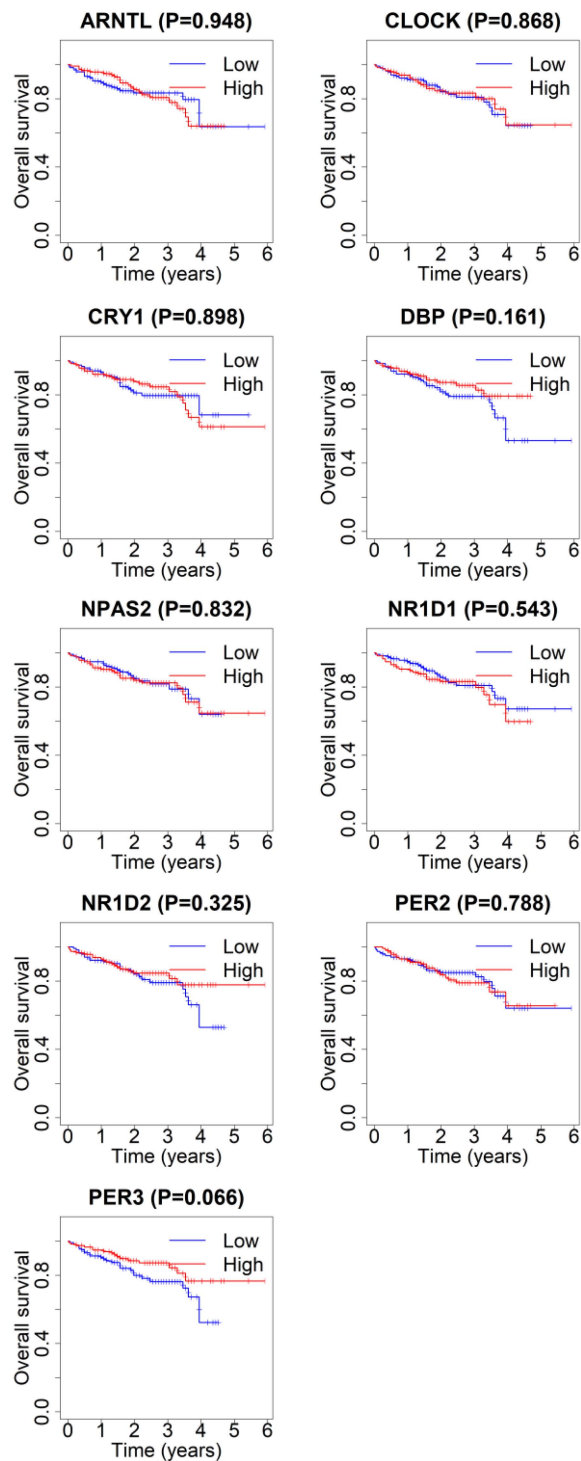

Supplement: Supplementary Materials — Supplementary material containing four figures is available on the publisher's website along with the published article. [file 8238833.f1.zip › FigS3.pdf]

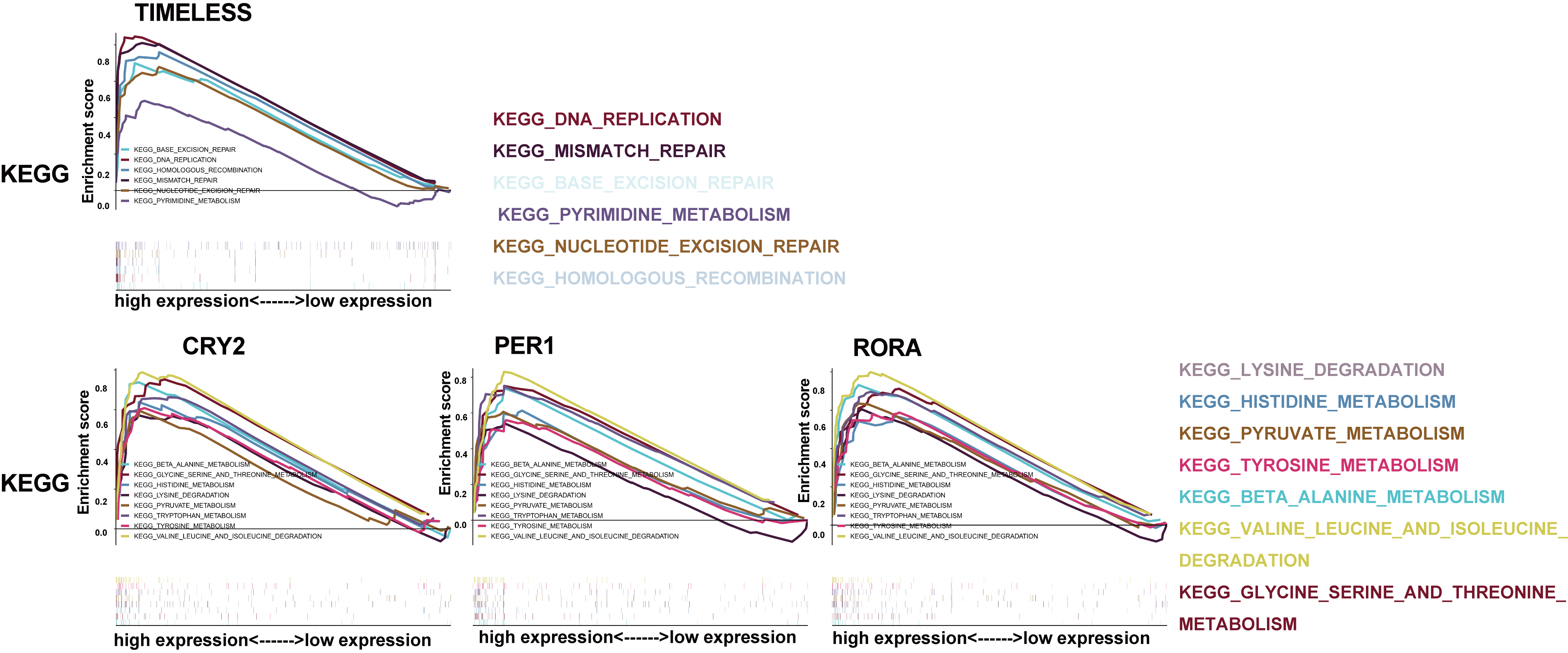

Supplement: Supplementary Materials — Supplementary material containing four figures is available on the publisher's website along with the published article. [file 8238833.f1.zip › figS4.tif]
